# Heterologous Expression of Mycobacterial Esx Complexes in *Escherichia coli* for Structural Studies Is Facilitated by the Use of Maltose Binding Protein Fusions

**DOI:** 10.1371/journal.pone.0081753

**Published:** 2013-11-29

**Authors:** Mark A. Arbing, Sum Chan, Liam Harris, Emmeline Kuo, Tina T. Zhou, Christine J. Ahn, Lin Nguyen, Qixin He, Jamie Lu, Phuong T. Menchavez, Annie Shin, Thomas Holton, Michael R. Sawaya, Duilio Cascio, David Eisenberg

**Affiliations:** 1 UCLA-DOE Institute for Genomics and Proteomics, University of California Los Angeles, Los Angeles, California, United States of America; 2 Department of Biological Chemistry, David Geffen School of Medicine at University of California Los Angeles, Los Angeles, California, United States of America; 3 Department of Chemistry and Biochemistry, University of California, Los Angeles, Los Angeles, California, United States of America; Centro Nacional de Biotecnologia – CSIC, Spain

## Abstract

The expression of heteroligomeric protein complexes for structural studies often requires a special coexpression strategy. The reason is that the solubility and proper folding of each subunit of the complex requires physical association with other subunits of the complex. The genomes of pathogenic mycobacteria encode many small protein complexes, implicated in bacterial fitness and pathogenicity, whose characterization may be further complicated by insolubility upon expression in *Escherichia coli*, the most common heterologous protein expression host. As protein fusions have been shown to dramatically affect the solubility of the proteins to which they are fused, we evaluated the ability of maltose binding protein fusions to produce mycobacterial Esx protein complexes. A single plasmid expression strategy using an N-terminal maltose binding protein fusion to the CFP-10 homolog proved effective in producing soluble Esx protein complexes, as determined by a small-scale expression and affinity purification screen, and coupled with intracellular proteolytic cleavage of the maltose binding protein moiety produced protein complexes of sufficient purity for structural studies. In comparison, the expression of complexes with hexahistidine affinity tags alone on the CFP-10 subunits failed to express in amounts sufficient for biochemical characterization. Using this strategy, six mycobacterial Esx complexes were expressed, purified to homogeneity, and subjected to crystallization screening and the crystal structures of the *Mycobacterium abscessus* EsxEF, *M*. *smegmatis* EsxGH, and *M. tuberculosis* EsxOP complexes were determined. Maltose binding protein fusions are thus an effective method for production of Esx complexes and this strategy may be applicable for production of other protein complexes.

## Introduction

Tuberculosis (TB) is the leading cause of death from a single infectious organism worldwide and *Mycobacterium tuberculosis* (Mtb), the causative agent of tuberculosis, is estimated to have infected one-third of the world's population [1]. A high number of small protein complexes are encoded within the Mtb genome and are believed to play critical roles in bacterial virulence and pathogenesis [2]–[4]. Of particular interest are the Esx complexes which are dimers of two subunits (ESAT-6, early secreted antigen of 6 kDa; and CFP-10, culture filtrate protein of 10 kDa) and which are secreted across the cytoplasmic membrane by Type VII secretion (T7S) systems [5]. Mycobacterial ESX secretion systems have been shown to be involved in a variety of physiological processes including conjugation in a non-pathogenic mycobacterium [6], [7], iron and/or zinc acquisition by both pathogenic and non-pathogenic mycobacterial species [8]–[10], and virulence of pathogenic mycobacteria [11], [12]. Consequently high resolution structural information is necessary to guide biochemical and biophysical studies of these protein complexes.

Structural studies of proteins, in general, are hampered by bottlenecks that exist at the key stages of production of soluble protein and protein crystallization [13], [14] and the study of protein complexes is further complicated by the requirement to produce two, or more, protein subunits in soluble form. Additional complications in the study of Esx protein complexes is that the majority of *M*. *tuberculosis* proteins expressed in *E*. *coli* are insoluble [15] and that expression of Mtb protein complexes may require a coexpression strategy to obtain folded, soluble protein complexes [16]. Strategies for coexpression of Mtb protein complexes in soluble form using *E*. *coli* expression systems are thus valuable in avoiding the use of other less well-characterized, and potentially cumbersome, bacterial expression systems and to avoid the need to explore laborious refolding and protein complex reconstitution schemes.

Certain highly soluble proteins have, when fused to target proteins, been demonstrated to promote the solubility, and in some instances, the folding of the proteins to which they are fused into biologically active form [17], [18]. Maltose binding protein (MBP) fusions have been demonstrated to be exceptional in this regard [19], [20], thus we evaluated the ability of MBP fusions to promote the solubility and folding of Esx complexes. Our results demonstrate that MBP fusions are an efficient approach for the production of Esx complexes and, in combination with intracellular proteolytic cleavage of the MBP fusion partner, allow production of Esx complexes in amounts and purity suitable for structural studies. Using this expression approach we expressed and purified six Esx complexes and determined the crystal structures of *M*. *abscessus* EsxEF (EsxEF_ma_), encoded by the MAB_3112 and MAB_3113 genes, *M*. *smegmatis* EsxGH (EsxGH_ms_), encoded by the MSMEG_0620 and MSMEG_0621 genes, and *M*. *tuberculosis* EsxOP (EsxOP_mt_), encoded by the Rv2346c and Rv2347c genes, at resolutions of 1.96 Å, 2.70 Å, and 2.55 Å, respectively.

## Materials and Methods

### Vector construction and Esx complex cloning

The pET28b (EMD Millipore, Billerica, MA) vector was used as the basis for the construction of expression vectors: pMA507, which contains an N-terminal hexahistidine tag (His_6_) followed by a tobacco etch virus (TEV) protease cleavage site; pMA510, which has an N-terminal MBP fusion followed by a His_6_ tag and TEV protease site; pMAPLe3, which allows intracellular processing of an N-terminal MBP fusion by TEV protease to yield a target protein with a C-terminal His_6_ tag; and pMAPLe4, which allows intracellular processing of an N-terminal MBP fusion by tobacco vein mottling virus (TVMV) protease to yield a target protein with a TEV protease cleavable N-terminal His_6_ tag. All enzymes were obtained from New England Biolabs (Ipswich, MA).

#### pMA507 and pMA510 vector construction

Briefly, pMA507 was constructed by inserting an oligonucleotide linker encoding the sequence MGSDKIGSHHHHHHENLYFQG between the NcoI and XhoI sites of pET28b. pMA510 was constructed by PCR-amplifying the MBP sequence from pMAL-C2 and inserting the PCR product between the NcoI restriction enzyme site and a BamHI restriction enzyme site encoded in the pMA507 linker such that amino acids 1–366 of mature processed *E*. *coli* MBP are inserted upstream of the His_6_ tag and TEV protease site. Both pMA507 and pMA510 contain the ccdB toxin gene downstream of the nucleotide sequence encoding the TEV protease site for use as a negative selection element in downstream cloning.

#### pMAPLe3 vector construction

For *in vivo* processing of MBP fusions the DNA sequence encoding amino acids 1–366 of mature processed *E*. *coli* MBP was PCR amplified using primers that incorporated a TEV protease site and His_6_ tag after the MBP sequence and the PCR product was inserted into the NcoI and XhoI sites of pET28b. The resulting MBP protein has the following sequence at its C-terminus: NSSSENLYFQSTHHHHHH where the underlined amino acids (ST) incorporate a ScaI restriction enzyme site. Two stop codons following the DNA sequence that encodes the His_6_ tag were encoded in the reverse primer. The gene encoding TEV protease and the promoter that drives its expression was PCR-amplified from pRK603 [21] and digested with the restriction enzymes PshAI and Tth111I whose recognition sequences were encoded in the primer extensions. This PCR product was digested with PshAI and Tth111I, treated with Klenow fragment to create blunt ends, phosphorylated with polynucleotide kinase, and ligated into the MBP containing vector which had been digested with PshAI to create pMAPLe2. To facilitate cloning into pMAPLe2, the ccdB toxin gene was PCR-amplified from pKM596 [22] using primers that incorporated ScaI restriction enzyme sequences in the primer extensions. The PCR product was digested with ScaI and inserted into the ScaI site of pMAPLe2 to make pMAPLe3. Protein complexes cloned into pMAPLe3 have seven amino acids (THHHHHH) appended to the C-terminus of the 3′ gene product and a single serine added to the N-terminus of the of the 5′ gene product after TEV cleavage of the MBP moiety.

#### pMAPLe4 vector construction

The TEV protease gene cassette in pMAPLe3 was replaced with the TVMV protease gene cassette, including the promoter that drives its expression, by the SLIC cloning technique using the plasmid pRK1037 [23] as the source of TVMV protease. The nucleotide sequence between the MBP gene and *ccd*B negative selection element was then modified by PCR-amplification using primers that introduced a TVMV protease sequence to generate pMAPLe4. The resulting MBP protein has the following sequence at its C-terminus: GSETVRFQSHHHHHHSSSENLYFQS. Fusion proteins expressed from this vector are cleaved intracellularly by the TVMV protease expressed from the same vector resulting in the cleaved partner proteins having the following sequence at their N-terminus: SHHHHHHSSSENLYFQS. TEV protease treatment of the partner protein following affinity purification results in the partner protein having a single serine residue present at its N-terminus.

#### Cloning of Esx complexes

Primer extensions contain 15 bp of sequence homologous to the flanking regions of the vector so that the PCR products could be inserted into the expression vectors using the SLIC cloning technique [24]. Primer sequences for Esx complexes cloned in this study are listed in Table S1. All PCR reactions were performed using Phusion DNA polymerase and PCR products were purified from agarose gels using the QIAquick gel extraction kit (Qiagen, Germantown, MD) and treated with T4 DNA polymerase following the SLIC protocol. For cloning into pMA507 and pMA510 the vectors were PCR-amplified with primers (PIPE.Vec.For. and PIPE.Vec.Rev.) homologous to either side of the site into which the Esx PCR products were to be inserted and the vector was purified and treated with T4 DNA polymerase in the same manner as the Esx PCR products. For pMAPLe3 and pMAPLe4, the vector was digested with ScaI restriction enzyme and the linearized vector treated with T4 DNA polymerase. PCR products of the Esx gene pairs (90 ng) was added to 45 ng of vector and the DNA mixture was transformed into *E*. *coli* DH5α (Invitrogen, Carlsbad, CA). The bicistronic operons encoding the Esx complex gene pairs [MSMEG_0620-MSMEG_0621 (EsxGH_ms_), *rv2346c*-*rv2347c* (EsxOP_mt_), *rv3444c*-*rv3445c* (EsxTU_mt_), and *rv3904c*-*rv3905c* (EsxEF_mt_)] were PCR-amplified from *Mycobacterium smegmatis* MC^2^155 and *M*. *tuberculosis* H37Rv genomic DNA and cloned into the pMA507, pMA510, and pMAPLe3 expression vectors. The bicistronic operons encoding the genes for three Esx complexes from *Mycobacterium abscessus* [EsxGH_ma_ (MAB_0665-MAB_0666), EsxEF_ma_ (MAB_3113-MAB_3112), and EsxTU_ma_ (MAB_3754c-MAB_3753c)] were PCR-amplified from *M*. *abscessus* genomic DNA and cloned into pMAPLe4. Positive transformants were identified by colony PCR using T7 and T7 terminator primers and the correct sequence of putative positive clones was verified by DNA sequencing (Genewiz, South Plainfield, NJ); the MAB_3112 and MAB_3113 genes were found to have eight mutations (MAB_3112: S23N, R37K, V75A, G93D; MAB_3113: P2A, T51A, E60Q, N77S) versus the protein sequences from the *M*. *abscessus* ATCC 19977 type strain.

### Small-scale expression and purification of Esx complexes

Expression and solubility of affinity tagged Esx complexes and their ability to bind Ni-NTA agarose was evaluated in 96 well format using a variation of the protocol of Klock *et al.* [25]. Briefly, *E*. *coli* BL21 (DE3) and/or *E*. *coli* Rosetta (DE3) harboring the expression plasmids were grown overnight in a 96 well block (Thomson Instrument Company, Oceanside, CA) at 37°C in 1 ml of LB media supplemented with 30 µg/ml kanamycin, and 34 µg/ml chloramphenicol for *E*. *coli* Rosetta (DE3), using a Shel Lab SI6R-HS shaking incubator with shaking at 650 rpm. The following day a 96 well block with 1 ml of fresh media supplemented with the appropriate antibiotics was inoculated with 50 µl of the overnight culture. The cultures were grown to an OD_600_ of 0.5 and protein expression induced by the addition of IPTG to a final concentration of 0.5 mM. The cultures were grown for an additional 4 hours at 37°C or, alternatively, were grown overnight at 18°C. Cultures were harvested by centrifugation and cell pellets were frozen and stored at −20°C pending lysis. The cell pellets were thawed and were resuspended in 500 µl lysis buffer A (20 mM HEPES, pH 7.5, 50 mM sucrose, 1 mM EDTA, 10 mM β-mercaptoethanol) supplemented with protease inhibitor cocktail (Sigma, St. Louis, MO), 1 mM PMSF, and ∼40 U/mL Ready-Lyse lysozyme (Epicentre, Madison, WI). The cell suspensions were shaken at 800 rpm for 20 minutes at 25°C and then MgCl_2_ was added to 5 mM and DNase I to 20 µg/ml. At this point 500 µl of lysis buffers B (50 mM HEPES, pH 7.8, 0.3 M NaCl), C (50 mM HEPES, pH 7.5, 1 M NaCl), or D (50 mM HEPES, pH 7.5, 0.3 M NaCl, 0.2% LDAO) were added to the cell lysate and the suspensions shaken for an additional 20 minutes. The lysates were clarified by centrifugation and the supernatants were transferred to a fresh block containing 50 µl of Ni-NTA agarose beads (Qiagen, Valencia, CA) and were incubated with shaking for one to two hours at 4°C. After incubation the suspensions were transferred to a 96-well filter plate and the beads were washed three times with 1 ml of wash buffer B (50 mM HEPES, pH 7.8, 0.3 M NaCl, 10 mM imidazole), C (50 mM pH 7.5, 1 M NaCl, 10 mM imidazole), or D (50 mM HEPES, pH 7.5, 0.3 M NaCl, 0.2% LDAO, 10 mM imidazole) prior to elution of the bound complexes by the addition of 100 µl of elution buffers B (50 mM HEPES, pH 7.8, 0.3 M NaCl, 0.3 M imidazole), C (50 mM pH 7.5, 1 M NaCl, 0.3 M imidazole), or D (50 mM HEPES, pH 7.5, 0.3 M NaCl, 0.2% LDAO, 0.3 M imidazole). Eluates were analyzed by SDS-PAGE using Criterion gels (Bio-Rad Laboratories, Hercules, CA).

### Large-scale expression and purification of Esx complexes

Selenomethionine(SeMet)-labeled EsxEF_ma_, EsxGH_ms_, and EsxOP_mt_ complexes were expressed as previously described [26] in *E*. *coli* BL21 (DE3) with the following modifications: the EsxEF_ma_ and EsxGH_ms_ cultures were grown for 18 hours at 18°C after induction of protein expression with 0.5 mM IPTG while the EsxOP_mt_ culture was grown under the same conditions but protein expression was induced with 1.0 mM IPTG. An unlabeled preparation of the EsxEF_ma_ complex was prepared using the same expression and purification conditions as for the SeMet-labeled EsxEF_ma_ complex with minor modifications as noted. The cells were harvested by centrifugation and the cell pellets were stored at −20°C pending lysis. Cell pellets were resuspended in lysis buffer (EsxEF_ma_: 20 mM Tris, pH 8.0, 300 mM NaCl, 10% glycerol; EsxGH_ms_ and EsxOP_mt_: 50 mM HEPES, pH 7.8, 150 mM NaCl) containing protease inhibitor cocktail (Sigma), 2 mM β-mercaptoethanol, 1 mM PMSF, DNase I (0.5 µg/ml), and lysozyme. The cells were lysed by sonication, and the lysates clarified by centrifugation (30,000 x g for 30 minutes at 4°C). The supernatants were incubated with Ni-NTA agarose beads (Qiagen, Valencia, CA) for one to two hours at 4°C and the suspension was then poured into a gravity column. The beads were washed twice with wash buffer (lysis buffer with 10 mM imidazole and for EsxGH_ms_ 10% glycerol was included), once with high salt buffer (same composition as wash buffer but with 1 M NaCl), again with wash buffer containing 50 mM imidazole, and the complexes then eluted with elution buffer (same composition as wash buffer but with 0.3 M imidazole). The complexes were further purified by size exclusion chromatography using a HiPrep 16/60 Sephacryl S-100 column (GE Healthcare, Piscataway, NJ) equilibrated in: 20 mM Tris, pH 8.0, 0.3 M NaCl, 10% glycerol (SeMet-labeled EsxEF_ma_), 20 mM Tris, pH 8.0, 150 mM NaCl, 10% glycerol, 2 mM β-mercaptoethanol (native EsxEF_ma_), 20 mM HEPES, pH 7.8, 150 mM NaCl, 10% glycerol, 2 mM β-mercaptoethanol (EsxGH_ms_), or 50 mM HEPES pH 7.8, 150 mM NaCl (EsxOP_mt_). The N-terminal His_6_ tag was cleaved from the native and SeMet-labeled EsxEF_ma_ complexes by TEV protease and the cleaved affinity tag and His_6_-tagged TEV protease separated from the cleaved target protein by passing the sample over Ni-NTA agarose beads prior to the size exclusion chromatography step. The SeMet-labeled EsxEF_ma_ complex was dialyzed into buffer IEX-A (20 mM Tris, pH 8.0, 150 mM NaCl, 10% glycerol) and further purified by ion exchange chromatography using a HiTrap Q HP column (GE healthcare) using a linear gradient from 0–100% buffer IEX-B (20 mM Tris, pH 8.0, 500 mM NaCl, 10% glycerol). The pure SeMet-labeled EsxEF_ma_ complex was subsequently dialyzed against storage buffer (20 mM Tris, pH 8.0, 0.3 M NaCl, 10% glycerol). The purified complexes were then concentrated for crystallization screening [EsxEF_ma_ (native): 11 mg/ml; EsxEF_ma_ (SeMet-labeled): 4.5 mg/ml; EsxGH_ms_: 27 mg/ml; EsxOP_mt_: 16 mg/ml].

### Crystallization of Esx complexes

All crystallization reactions were performed using the hanging drop vapor diffusion method at 18°C. Crystals of the EsxEF_ma_ native complex were grown by mixing protein stock solution 1∶2 with reservoir solution (0.1 M tri-sodium citrate, pH 5.6, 0.2 M potassium sodium tartrate, 2 M ammonium sulfate) while the SeMet-labeled EsxEF_ma_ crystals were grown by mixing the protein solution 1∶1 with reservoir solution (1.8 M tri-ammonium citrate, pH 7.0). Irregular “coffee bean” shaped crystals of the native EsxEF_ma_ complex grew in 1 week while “spearhead” shaped crystals of the SeMet-labeled EsxEF_ma_ complex grew in 4–6 weeks. Native EsxEF_ma_ crystals were cryo-protected in reservoir solution containing 25% glycerol and SeMet-labeled crystals were flash frozen without any additional manipulation. Hexagonal rod crystals of the EsxGH_ms_ complex were grown in 2 months by mixing the protein stock solution 1∶1 with reservoir solution (460 mM potassium sodium tartrate, 35% glycerol, 95 mM HEPES, pH 7.5) and were mounted directly from the crystallization drop. Crystals of EsxOP_mt_ in crystal form I were grown by mixing protein stock solution 2∶1 with reservoir solution (9% isopropanol, 90 mM sodium acetate trihydrate pH 4.6, 200 mM CaCl_2_). Hexagonal rod shaped crystals of this complex grew in 2–3 weeks and were cryoprotected with paraffin oil. Trapezoidal prism crystals of EsxOP_mt_ (form II) were grown by mixing complex at 8 mg/ml in storage buffer (20 mM HEPES, pH 7.8, 150 mM NaCl, 2 mM ZnSO_4_, 38 mM β-mercaptoethanol) 1∶1 with reservoir solution (11% PEG 3350, 40 mM citric acid pH 3.5). Crystals of EsxOP_mt_ (form II) grew to full size in 2–3 weeks and were cryoprotected using paraffin oil.

### Data collection and structure determination

Diffraction data were collected on beamlines 24-ID-C and 24-ID-E at the Advanced Photon Source of Argonne National Lab. Diffraction data for EsxEF_ma_ and EsxOP_mt_ (form II) were processed with XDS [27] while data for EsxGH_ms_ and EsxOP_mt_ (form I) were processed and scaled with DENZO and Scalepack [28]. The position of the selenium sites for the EsxEF_ma_ and EsxGH_ms_ substructures were found with HKL2MAP [29] while the selenium sites in the EsxOP_mt_ (form I) substructure were determined using Phenix [30]. Initial models were built with Phenix AutoBuild [30] and subsequent model building was performed manually using Coot [31]. The unlabeled structures of EsxEF_ma_ and EsxOP_mt_ (form II) were solved by molecular replacement using the program phenix.phaser [30] using the models obtained from the respective SeMet-labeled crystals. Refinement of EsxEF_ma_ and EsxOP_mt_ (forms I and II) structures was performed with Phenix.refine [30] and the EsxGH_ms_ structure was refined with Refmac [32]. Figures were prepared with PyMOL (), structural homology searches were performed with DALI [33], electrostatic surface potentials were calculated with APBS [34], solvent-accessible surface area was calculated with PISA [35], shape complementarity was calculated with *SC* [36], and mapping of sequence identity onto molecular surfaces was performed with the ConSurf server [37] using sequence alignments generated with Clustal Omega [38]. Unless otherwise mentioned the following structures and chains were used for the calculations described above: EsxEF_ma_, PDBid 4IOX, chains A and B; EsxGH_ms_, PDBid 3Q4H, chains A and B; EsxOP_mt_, PDBid 4GZR, chains C and D. Protein identifiers for protein sequences used to generate sequence alignments and ConSurf calculations are listed in Table S2. The coordinates and molecular structure factors for the Esx complex structures determined in this study have been deposited in the Protein Data Bank (http://www.rcsb.org) under the accession codes: 4IOX (EsxEF_ma_), 3Q4H (EsxGH_ms_), 3OGI (EsxOP_mt_, form I), and 4GZR (EsxOP_mt_, form II).

## Results

### Expression of mycobacterial Esx complexes

**Figure 1:**
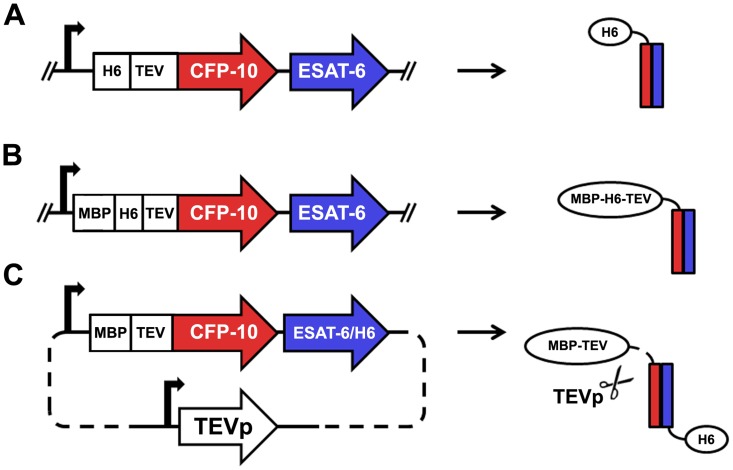
Schematic of Esx complex coexpression strategies. Bicistronic operons encoding the Esx complex genes, separated by a naturally occurring intergenic region of variable length, were cloned into three different expression vectors. (A) The two subunits are coexpressed from a single bicistronic transcript with an N-terminal His_6_ tag and TEV protease site on the CFP-10 homolog. (B) The two subunits are coexpressed from a single bicistronic transcipt with an N-terminal MBP fusion with His_6_ tag and TEV protease site on the CFP-10 homolog. (C) The two subunits are coexpressed from a single bicistronic transcript with an N-terminal MBP fusion with TEV protease site on the CFP-10 homolog and a C-terminal His_6_ tag on the ESAT-6 homolog. Concurrent expression of TEV protease (TEVp) cleaves the MBP moiety from the CFP-10 homolog intracellularly at the TEV protease site positioned between the MBP C-terminus and CFP-10 N-terminus.

Four mycobacterial Esx complexes were cloned using the native bicistronic operon structure which has the CFP-10 homolog subunit separated from the downstream ESAT-6 homolog by an intergenic region of variable length and sequence (Table S3). The Esx operons were cloned into *E*. *coli* expression vectors (Figure 1) that allowed expression of the complexes with either: 1, an N-terminal His_6_ tag and TEV protease cleavage site on the CFP-10 homolog (pMA507); 2, an MBP fusion protein, His_6_ tag and TEV protease cleavage site on the N-terminus of the CFP-10 homolog (pMA510); or 3, with a C-terminal His_6_ tag on the ESAT-6 homolog and an N-terminal MBP fusion and TEV protease site on the CFP-10 homolog that is cleaved intracellularly by concurrent expression of TEV protease during recombinant protein expression (pMAPLe3). To simplify the cloning process we chose to express the Esx complexes from a single bicistronic message as our previous experience with Mtb protein complexes (our unpublished results) and that of others [39] has shown that *E*. *coli* recognizes the *M*. *tuberculosis* operon structure and can produce two proteins from a single bicistronic transcript.

Maltose binding protein is a potent solubilizing fusion partner and is capable of solubilizing misfolded proteins [40] thus an assay is required to ascertain whether the protein of interest exists in a folded or unfolded state. Esx complexes lack a defined assayable enzymatic function so small-scale affinity purification was used to assess whether the expressed subunits of the Esx complexes were soluble and properly folded. We reasoned that the ability of the untagged subunit to copurify with the His_6_-tagged subunit indicates that both complex subunits are properly folded and that the proper dimeric complex has been formed. Small-scale affinity purification screening is particularly well suited for structural genomics projects in that the conditions under which a complex is soluble and can be purified are rapidly determined. However, it does not determine the relative expression and solubility levels of the individual components of the complex. The small-scale affinity purification results are summarized in Table S4. Large-scale protein purification that includes a size exclusion chromatography step was subsequently used to validate the small-scale affinity purification results.

**Figure 2:**
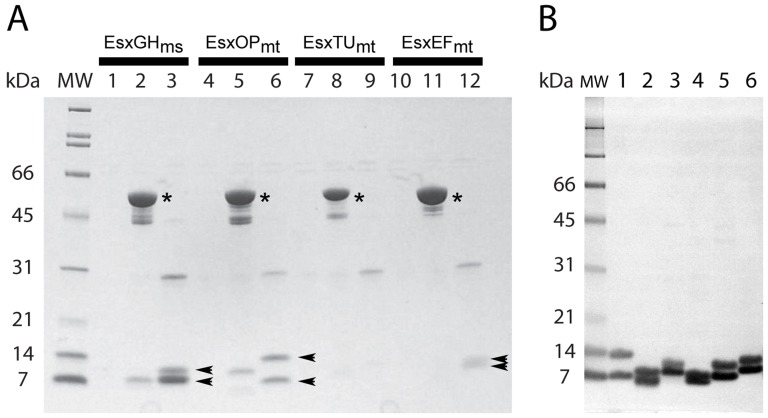
Expression and affinity purification of mycobacterial Esx complexes. (A) SDS-PAGE analysis (Any-kD TGX gel, Bio-Rad) of small-scale expression and affinity purification of His_6_–tagged proteins: lanes 1–3, EsxGH_ms_ complex; lanes 4–6, EsxOP_mt_ complex; lanes 7–9, EsxTU_mt_ complex; and lanes 10–12, EsxEF_mt_ complex. The first lane for each complex is expression of the complex from the pMA507 vector with the His_6_ tag alone on the N-terminus of the CFP-10 homolog, the second lane is the complex expressed from pMA510 which expresses the CFP-10 homolog with an N-terminal MBP-His_6_ fusion (indicated by an asterisk), and the third lane is the complex expressed from the pMAPLe3 vector which allows proteolytic cleavage of the MBP moiety *in vivo* and purification of the complex via a C-terminal His_6_ tag on the ESAT-6 homolog. Arrows indicate the presence of Esx complex subunits. (B) SDS-PAGE analysis (Any-kD TGX gel, Bio-Rad) of concentrated Esx complex samples from large-scale purification (∼3 µg per lane): 1, EsxOP_mt_; 2, EsxEF_mt_; 3, EsxGH_ms_; 4, EsxGH_ma_; 5, EsxEF_ma_; and 6, EsxTU_ma_.

When the Esx complexes were expressed with a His_6_ tag on the CFP-10 subunit alone none of the four tested Esx complexes were purified by small-scale affinity purification (Figure 2, lanes 1, 4, 7, and 10). With extensive expression optimization we were able to obtain expression of the complexes in soluble form but in trivial amounts insufficient for biochemical characterization (data not shown). This result is expected as the majority of *M*. *tuberculosis* proteins expressed in *E*. *coli* are insoluble [15]. Moreover, our experience with *M*. *tuberculosis* PE-PPE complexes [16] has shown that even with a coexpression strategy Mtb protein complexes are often insoluble when expressed in *E*. *coli*.

The ability of MBP fusions to promote folding of fused passenger proteins was apparent when we expressed the CFP-10 subunits with an N-terminal MBP fusion. When the CFP-10 homologs were expressed as MBP fusions using the pMA510 vector we found that in all four test cases the CFP-10 homologs were successfully expressed as soluble MBP fusions (Figure 2, lanes 2, 5, 8, and 11). However, in only two instances were the cognate ESAT-6 homolog protein partners copurified using this strategy (Figure 2, lanes 2 and 5). The second MBP fusion protein strategy employed intracellular processing of the MBP fusion proteins to evaluate whether the passenger proteins would be soluble after cleavage of the MBP molecule [21], [41]. The expression vector used in this approach (pMAPLe3) was engineered to allow simultaneous coexpression of TEV protease to cleave passenger proteins from the MBP moiety *in vivo* via a TEV protease site positioned between MBP and the CFP-10 passenger protein. This approach allowed three of four Esx complexes to be successfully expressed in soluble form and affinity purified via the C-terminal His_6_ tag on the ESAT-6 subunit (Figure 2, lanes 3, 6, and 12).

### Crystallization and structure determination of mycobacterial Esx complexes

The Esx complexes found to be expressed and soluble in the small-scale screen using the intracellular processing method were grown in large-scale and purified to homogeneity by affinity and size exclusion chromatography (Figure 2B). The relative amounts of purified protein obtained from large-scale purification (EsxGH_ms_, ∼8 mg/L; EsxOP_mt_, ∼12 mg/L; and EsxEF_mt_, ∼2.5 mg/L) were consistent with the yields from the small-scale screen. Three additional Esx complexes (EsxGH_ma_, EsxEF_ma_, and EsxTU_ma_) from *M*. *abscessus*, a fast-growing pathogen, were cloned into pMAPLe4, an expression vector similar to pMAPLe3 with the exception that the MBP moiety is cleaved from the CFP-10 passenger by TVMV protease leaving a TEV protease cleavable His_6_ tag on the N-terminus of the CFP-10 subunit. The substitution of an N-terminal cleavable tag on the CFP-10 subunit in lieu of the C-terminal His_6_ tag on the ESAT-6 subunit was deemed to be more amenable to generating a well ordered crystal lattice as the number of potentially disordered amino acid residues is reduced. The three *M*. *abscessus* Esx complexes were found to be soluble using our small-scale expression and purification screening method and were subsequently purified in large-scale in quantities suitable for crystallization screening (Figure 2B).

The six Esx complexes purified in large-scale were subjected to extensive crystallization screening and strongly diffracting crystals were obtained for EsxEF_ma_, EsxGH_ms_, and EsxOP_mt_. The structures of these complexes were determined by single wavelength anomalous diffraction (EsxEF_ma_ and EsxGH_ms_) and multiple wavelength anomalous diffraction (EsxOP_mt_) at resolutions of 3.0 Å, 2.70 Å, and 2.55 Å, respectively. A crude low resolution model of the SeMet-labeled EsxEF_ma_ complex was used to obtain a molecular replacement solution for a high resolution (1.96 Å) EsxEF_ma_ native dataset and a second crystal form of EsxOP_mt_, solved by molecular replacement using the EsxOP_mt_ model from form I, at 2.55 Å was also obtained. Diffraction data, refinement statistics, and model contents are summarized in Tables 1 and 2.

**Table 1:** Data collection statistics for *M*. *abscessus* EsxEF, *M*. *smegmatis* EsxGH, and *M*. *tuberculosis* EsxOP complexes.

	EsxEF_ma_ (native)	EsxEF_ma_ (SeMet)	EsxGH_ms_	EsxOP_mt_ (form I)		EsxOP_mt_ (form II)
Beamline	24-ID-C	24-ID-C	24-ID-E	24-ID-C		24-ID-C
**Crystal Parameters:**				Peak	Inflection	
Space group	P1	P3_1_	P6_3_	P3_2_21	P3_2_21	C222_1_
Wavelength (Å)	0.9791	0.9793	0.9792	0.9792	0.9794	1.282
Unit cell constants (Å) at -173°C	47.6 74.1 84.4 (114.6° 103.3° 95.5°)	46.4 46.4 71.4 (90° 90° 120°)	105.6 105.6 71.3 (90° 90° 120°)	66.4 66.4 162.6 (90° 90° 120°)	66.4 66.4 162.5 (90° 90° 120°)	76.1 112.1 95.3 (90 90 90)
**Data Quality:**						
Resolution range [Å]*	20.00–1.96 (2.01–1.96)	30.00–3.00 (3.08–3.00)	80.00–2.70 (2.77–2.70)	28.31 – 2.55 (2.75–2.55)	100.00–2.55 (2.64–2.55)	52.53–2.55 (2.62–2.55)
Mean redundancy	1.96	2.98	3.70	5.50	5.55	5.56
Number of unique reflections	67097	6796	46063	25291	25637	13340
Completeness of data [%]*	93.0 (90.0)	98.5 (99.5)	99.0 (99.0)	96.5 (100)	98.3 (100)	98.1 (95.3)
Mean I/*σ*(*I*)	13.7	7.5	8.0	19.9	15.4	14.9

* Numbers in parentheses refer to the high resolution data shell.

**Table 2:** Refinement statistics for *M*. *abscessus* EsxEF, *M*. *smegmatis* EsxGH, and *M*. *tuberculosis* EsxOP complexes.

	EsxEF_ma_ (native)	EsxGH_ms_	EsxOP_mt_ (form I)	EsxOP_mt_ (form II)
PDB Accession	4I0X	3Q4H	3OGI	4GZR
**Refinement Residuals:**				
Space group	P1	P6_3_	P3_2_21	C222_1_
Resolution range [Å]*	20.00–1.96 (1.98–1.96)	80.00–2.70 (2.77–2.70)	28.31 – 2.55 (2.75–2.55)	38.05–2.55 (2.64–2.55)
R*work* [%[*	15.9 (26.6)	21.0 (24.1)	21.6 (27.7)	19.9 (25.2)
R*free* [%]*	21.3 (33.3)	26.8 (27.7)	26.0 (31.5)	23.3 (28.6)
Test set size [%], selection	10, random	5, random	5, random	10, random
**Model Quality:**				
RMSD Bond lengths [Å]	0.012	0.008	0.008	0.014
RMSD Bond angles [°]	1.16	0.87	1.07	1.23
Ramachandran plot (%)				
Most favored	98.4	97.1	94.9	96.7
Additionally allowed	1.6	2.5	4.3	3.3
Generously allowed	0	0	0.9	0
Disallowed	0	0.3	0	0
**Model Contents:**				
Protein residues	EsxE_ma_: A2–A76, C2–C76, E3–E69, G2–G69, I1–I76, K2–78; EsxF_ma_: B8–B90, D9–D91, F7–F45/F51–F96, H12–H87, J10–J46/J50–J88, L9–L90	EsxG_ms_: A5–A94, C5–C91; EsxH_ms_: B7–B86, D1–D83.	EsxO_mt_: A13–A39/A49–A69, C13–C68; EsxP_mt_: B5–B41/B46–B97, D7–D38/D56–D97	EsxO_mt_: A12–A69; C9–C69; EsxP_mt_: B4–B38/B54–B95, D3–D38/D51–D94
Waters	337	33	35	31
Ligands	SO_4_^2−^ (1), glycerol (1), β-mercaptoethanol (1)	–	–	SO_4_^2−^ (1)

* Numbers in parentheses refer to the high resolution data shell.

### Structures of mycobacterial EsxEF_ma_, EsxGH_ms_, and EsxOP_mt_ complexes

**Figure 3:**
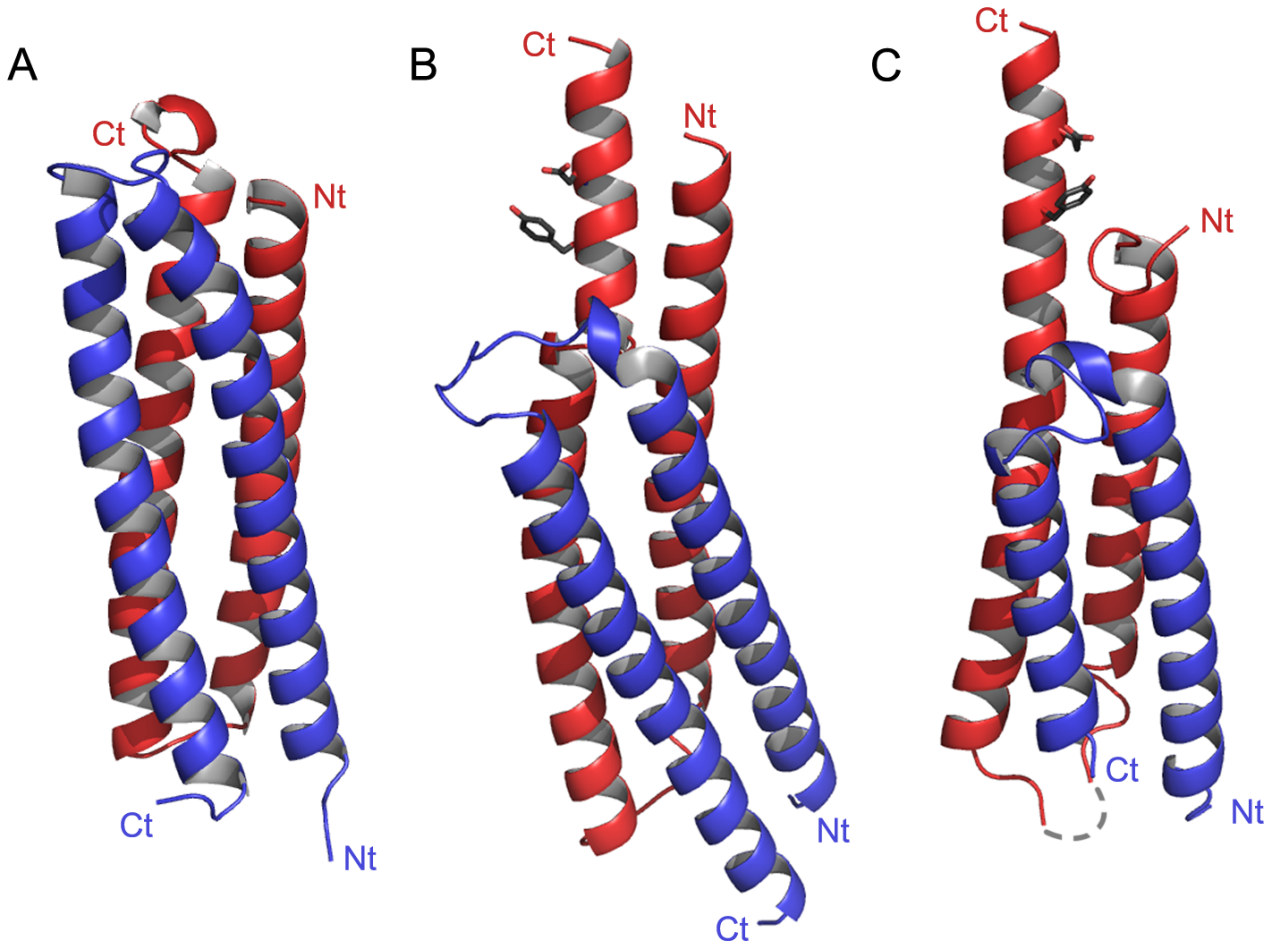
Ribbon representations of the structures of the Esx complexes determined in this study. The CFP-10 homologs are colored red and the ESAT-6 homologs are colored blue. (A) the EsxEF_ma_ complex; (B), the EsxGH_ms_ complex; and (C), the EsxOP_mt_ complex. The N- and C-termini of individual chains are labeled and the disordered loop region of EsxO_mt_ that connects its two α-helices is indicated by a dashed line. The tyrosine and acidic residues of the secretion signals of the EsxGH_ms_ and EsxOP_mt_ complexes are shown in stick representation.

The asymmetric units for the EsxEF_ma_ crystal forms have a single complex in the asymmetric unit for the SeMet-labeled protein and six complexes in the asymmetric unit of the native protein crystal form while the EsxGH_ms_ and EsxOP_mt_ (both crystal forms) contain two heterodimeric complexes. The overall architecture of the complexes is similar to known Esx structures [9], [26], [39], [42] in that each heterodimer forms a four helix bundle with the CFP-10 and ESAT-6 subunit homologs each contributing an α-helical hairpin to the complex (Figure 3). The α-helices of each subunit are connected by a flexible loop containing the canonical ESAT-6/CFP-10 signature motif (Trp-Xaa-Gly; WXG) although the EsxG_ms_ CFP-10 homolog contains a modified motif (His-Xaa-Gly). The WXG-containing loops are well ordered in the EsxGH_ms_ complexes and in most of the EsxEF_ma_ complexes but the majority of the connecting loops are disordered in the EsxOP_mt_ subunit structures (Table 2). Those connecting loops which were found to be ordered have, as in existing Esx structures, the tryptophan sidechain of the WXG motif buried in the subunit interface. Additional regions of disorder are found at the N- and C-termini of the complexes with the EsxF_ma_ and EsxO_mt_ subunits having a substantial number of disordered residues at their C-termini.

The subunit interfaces in the four-helix bundles of the Esx complexes are similar to those of the previously determined Esx complex structures in that the intermolecular interface between the CFP-10 and ESAT-6 subunits is largely hydrophobic. The average amount of buried surface area in the subunit interfaces varies considerably [EsxEF_ma_, ∼1605 Å^2^; EsxGH_ms_, ∼1555 Å^2^; EsxOP_mt_ (form I), ∼1025 Å^2^; and EsxOP_mt_ (form II), ∼1175 Å^2^] and the percentage of the surface of each subunit buried in the complex interface is also highly variable, from 13.5–33%, and is related to the degree to which the termini of the subunits are ordered and to the conformations that they adopt. Shape complementarity (*Sc*) statistics of 0.77 for EsxEF_ma_, 0.74 for EsxGH_ms_, and 0.69 and 0.71 for EsxOP_mt_ forms I and II, respectively, indicate that the interfaces are highly complementary, with biologically significant interfaces such as antibody-antigen interactions generally having values in the range of 0.65–0.85. The complexes are further stabilized by hydrogen bonds and salt bridges.

## Discussion

The heterologous expression of proteins in soluble form is a major bottleneck in structural studies and is particularly problematic for expression of Mtb proteins. A recent analysis of target status data for mycobacterial protein targets (http://www.webtb.org/Targets/), collected by the Tuberculosis Structural Genomics Consortium (TBSGC), found only 48% of Mtb proteins expressed in *E*. *coli* are soluble. In comparison, two studies of homologous overexpression of His_6_-tagged *E*. *coli* proteins in *E*. *coli* expression hosts found that 60% and 92.5% of the targets were soluble when expressed in small-scale format [43], [44]. However, in the latter study only 73% of the targets were found to be soluble using large-scale expression and purification methods. Data from the Northeast Structural Genomics Consortium (http://www.spine.nesg.org) found 60% of 697 *E*. *coli* targets were expressed in soluble form although this number does not provide statistics on construct engineering approaches targeting individual domains or truncations of constructs to improve solubility. The TBSGC data for mycobacterial species (for which a significant number of target statuses are reported) other than Mtb finds that heterologous expression of target proteins from *M*. *abscessus*, *M*. *avium*, *M*. *marinum*, and *M*. *avium* subsp. *paratuberculosis* produces soluble protein at a similar rate (56–61%) as for homologous overexpression of *E*. *coli* proteins. The remaining mycobacterial species are at opposite end of the spectrum in terms of heterologous production of soluble proteins with only 40% of *M*. *leprae* proteins expressed in soluble form while over 72% of heterologously expressed *M*. *smegmatis* MC2 155 proteins were found to be soluble.

The failure of a protein to express in soluble form in a heterologous expression host is unlikely to be attributable to a single cause as multiple factors at many levels influence protein solubility. High GC-content and secondary structure in the mRNA 5′ region has been shown to have a significant deleterious effect on protein expression levels [45] suggesting that the 66% GC-content of the Mtb genome, relative to 51% for *E*. *coli*, could be partially responsible for the insolubility of heterologously expressed Mtb proteins. However, the GC-content of other mycobacterial genomes is similar to Mtb and the TBSGC data (www.webtb.org/Targets) shows that proteins from these species, with the exception of *M*. *leprae*, are expressed in soluble form almost as often as homologously expressed *E*. *coli* proteins. Thus GC-content alone is not the sole factor responsible for the poor solubility of heterologously expressed Mtb and *M*. *leprae* proteins.

Protein solubility is also affected by differences in organism-specific codon usage (codon usage bias) between the heterologous expression host and the source of the gene to be expressed [46]. Codon usage has a substantive effect on co-translational protein folding by altering the rate at which the nascent polypeptide is synthesized [47]. Rare, or slow, codons are present in specific regions of mRNA messages and function to alter translation rates, and thus protein folding, in mRNA segments that correspond to protein domain boundaries [48], [49] or in the transition from unstructured to structured regions of the nascent polypeptide chain [50], allowing structural elements additional time to fold. As a result of differences in codon usage bias the relative position of rare codons within a target gene in regard to structural elements is likely to be altered versus the expression host. As a result protein translation rates are likely to be significantly altered with a potential negative effect on protein solubility. Attempts to manipulate the expression of heterologous proteins by mitigating the effects of rare codons, through the use of synthetic genes or accessory plasmids encoding tRNA molecules for rare codons, have had mixed results. The increased translation rates associated with these strategies appear to uncouple the co-translational process of chain elongation and protein folding leading to high amounts of misfolded, insoluble protein [47], [51].

The physicochemical properties of the proteins themselves are also an important determinant of protein solubility. An analysis of data from large-scale structural genomics centers found the proteins most likely to be successfully expressed were of shorter length, had lower hydrophobicity, and were moderately acidic [52]. The results from large-scale structural genomic centers may be skewed as the methodology employed by the centers may tend to favor production of proteins with these particular characteristics. Additional complicating factors in heterologous protein expression are the potential for organism-specific chaperones and post-translational protein modifications. Goldstone *et al*. found that five of eight Mtb proteins that were insoluble when expressed in *E*. *coli* were soluble when expressed in *M*. *smegmatis*, a close relative of *M*. *tuberculosis* [53]. The authors speculate that mycobacterial chaperones may be responsible for the increase in target protein solubility, but there are many other factors, including the effects of codon usage bias, which may be responsible for the dramatic increase in protein solubility.

The difference in success rate between different mycobacterium for the heterologous production of target proteins in soluble form is unusual. One would expect that the relatedness of mycobacterial species and similar GC-content would result in a similar success rate for the heterologous production of target proteins in soluble form. A possible reason for the discrepancy is that target selection for strains other than Mtb and *M*. l*eprae* may be biased towards targets that have a greater chance of being expressed in soluble form. Structural genomics efforts investigating mycobacterial protein structures have concentrated on Mtb with over 2200 unique proteins having been targeted (http://www.webtb.org/Targets/) while the other mycobacterium represented in the TBSGC data have only 170–370 targets per species. As more data is accumulated the question of the solubility of heterologously expressed mycobacterial proteins may be better answered.

### MBP fusions promote Esx complex expression

We used a small-scale affinity purification screen to evaluate the ability of maltose binding protein fusions to produce soluble, folded Esx complexes. Coexpression of the ESAT-6 and CFP-10 subunits homologs without an N-terminal MBP fusion on the CFP-10 subunit failed to produce any of the four test complexes in yields sufficient for structural studies. This result is consistent with our past experiences demonstrating that coexpressing the two subunits of a complex is not necessarily sufficient for the production of Mtb protein complexes in soluble form in *E*. *coli* [16]. In contrast, the use of N-terminal MBP fusions to the CFP-10 homologs allowed three of the four tested Esx complexes to be expressed and purified in quantities suitable for structural studies.

The ability of MBP to facilitate production of recalcitrant mycobacterial proteins was evident in our first MBP fusion strategy in which all four CFP-10 homologs were successfully expressed and purified as soluble N-terminal MBP fusions. However, in only two of four instances were both subunits of the heteroligomeric Esx complex copurified using this strategy (Figure 2). Multiple explanations are possible for the failure of the ESAT-6 homologs to copurify with their cognate CFP-10 partners: 1, the ESAT-6 homolog may not be expressed; 2, the ESAT-6 subunit may be insoluble; 3, the CFP-10 subunit may be unable to fold into a conformation allowing productive complex formation; and 4, the transient physical interactions between MBP and its fused passenger protein, believed to prevent aggregation of the passenger protein [54], may prevent interaction of the CFP-10 molecule and its natural ESAT-6 protein partner. A limitation of screening for soluble complexes is that it does not distinguish amongst these possibilities and specifically whether the untagged subunit has been expressed in either soluble or insoluble form.

In our second MBP fusion strategy we employed intracellular processing of the MBP fusion proteins to evaluate whether the passenger proteins would be soluble after cleavage of the MBP molecule [21], [41]. As many proteins are insoluble after cleavage of the MBP moiety [55], [56] this strategy determines whether MBP fusions are a viable experimental strategy for producing the passenger protein(s) in soluble form. Interestingly, while the EsxEF_mt_ complex was expressed and purified using this strategy (Figure 2, lane 12) the dimeric complex was not purified using the unprocessed MBP fusion vector (Figure 2, lane 11). This suggests that the failure to copurify the ESAT-6 homologs for the EsxTU_mt_ and EsxEF_mt_ complexes in the absence of intracellular MBP cleavage may be attributable to two distinct pathways. In both instances MBP acts in its “holdase” capacity [57] maintaining its passenger, the CFP-10 homolog, in a soluble state. In the first instance, of EsxT_mt_, the passenger protein is likely not properly folded. While in the second instance, of EsxE_mt_, the passenger appears to fold productively but interactions with the ESAT-6 homolog (EsxF_mt_) may be physically prohibited by either MBP-EsxE_mt_ interactions or by MBP impeding the EsxE_mt_-EsxF_mt_ interactions required for complex formation. However, an alternative explanation is that the presence of the C-terminal His_6_ tag on the EsxF_mt_ subunit, derived from the pMAPLe3 vector, increases the solubility of this subunit thus allowing the complex to form.

While our MBP fusion protein strategy for production of protein complexes is straightforward and time efficient, we did fail to purify the EsxTU_mt_ complex. The screen for soluble protein complexes is particularly well-suited for structural genomics approaches targeting the most tractable targets. However, a major limitation of the screen is that it does not fully address the expression and solubility of the individual subunits and fails to identify conditions under which a component of the complex is expressed but is insoluble. As a result it is difficult to draw general conclusions about the expression and/or solubility of individual components of the complex under different experimental conditions. High value targets should thus be subjected to alternate salvage pathways to determine conditions that produce both complex subunits in soluble form. For mycobacterial protein complexes, in particular, use of the *M*. *smegmatis* expression system is a likely next step as the EsxTU_mt_ and the EsxEF_mt_ complexes were both successfully produced using this system [39].

### Esx signal sequences adopt multiple conformations

The extent of disorder present at the N- and C-termini of both subunits of our mycobacterial Esx complex structures is not unprecedented as the structures of the EsxAB_mt_ [39], [42], EsxGH_mt_ [9], and EsxRS_mt_ [26] complexes have considerable amounts of disordered residues (between 4–20 amino acids) at the N- and C-termini of both complex subunits. Disordered subunit termini may mediate protein-protein interactions between Esx complexes and other proteins and there is evidence demonstrating the interaction of the ESAT-6 (EsxA_mt_) C-terminus with surface receptors of host immune system cells [58]. Additional evidence implicates the C-terminus of CFP-10 homologs in protein-protein interactions as the CFP-10 C-terminus contains a signal sequence directing secretion of the complex across the cytoplasmic membrane [59] and mutation of either of two conserved amino acids in an amino acid motif (Tyr-Xaa-Xaa-Xaa-Glu/Asp) located within this region abolishes secretion [60].

The C-termini of the CFP-10 homolog subunits of our EsxGH_ms_ and EsxOP_mt_ (forms I and II) structures, which contain the secretion motif, are ordered and adopt helical structures; in the EsxOP_mt_ structure the secretion motif containing helix is an extension of the C-terminal helix of the α-helical hairpin while in EsxGH_ms_ the secretion motif is primarily contained in a short, 13 amino acid, helix linked to the α-helical hairpin by a short turn. The sidechains of the conserved amino acids in the secretion motif are oriented outward and away from the bundle in the EsxGH_ms_ structure while in the EsxOP_mt_ structure the sidechains are oriented towards the center of the four helix bundle (Figure 3). The crystal structure of *M*. *tuberculosis* CFP-10 (EsxB_mt_; PDBid 3FAV-chain C) also has a partially ordered C-terminus and in this instance the side chains of the conserved amino acids of the secretion motif are in a helical region with the sidechains pointing to the central axis of the helical bundle. While crystal packing is most likely responsible for stabilizing these regions in different helical conformations, it is likely that the helical conformation is biologically relevant. Indeed, the N-terminal Type I signal sequences of proteins exported by the general secretory (Sec) system have been shown to adopt helical conformations in the presence of lipids or upon association with components of the Sec machinery [61], [62]. The helical structure of the C-termini of multiple CFP-10 homologs and the i+4 spacing of the T7S secretion motif that positions the sidechains of critical amino acid residues on the same face of a helix both suggest that this conformation facilitates a specific protein-protein interaction, likely with another component of the T7S system secretion machinery.

### Features of the Esx complex surfaces

**Figure 4:**
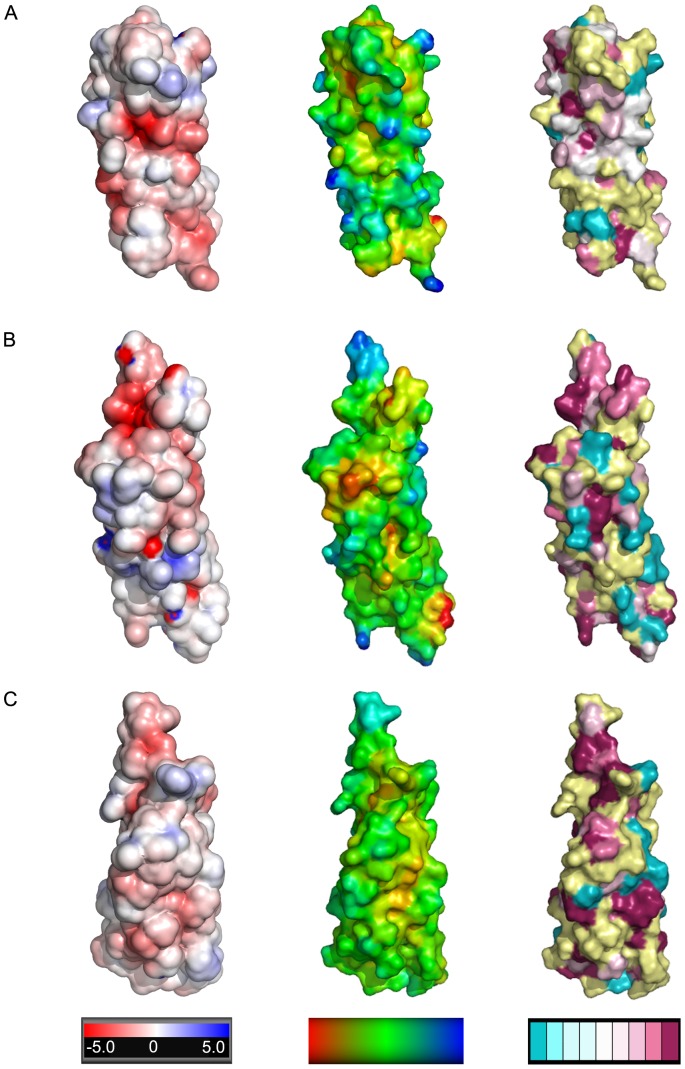
Surface characteristics of Esx complexes (I). The complexes are shown in the same orientation as in Figure 3 with the ESAT-6 homolog subunit facing the viewer. (A) the EsxEF_ma_ with the surface colored by electrostatic potential (first column), hydrophobicity (second column), and sequence identity (third column). (B) the EsxGH_ms_ complex colored as in (A). (C) the EsxOP_mt_ complex colored as in (A). Colored bars under each column indicate: column 1, electrostatic surface potentials of +/− 5 kT calculated at an ionic strength of 150 mM; column 2, hydrophobicity with a gradient of red (most hydrophobic) to blue (least hydrophobic); and column 3, the degree of sequence conservation with variable regions in teal, highly conserved regions in burgundy, and the regions where the degree of conservation could not be assigned with confidence in yellow. Sequence conservation was calculated using alignments of 15 (EsxEF_ma_), 21 (EsxGH_ms_), and 27 (EsxOP_mt_) pairs of homologous sequences (listed in Table S2).

**Figure 5:**
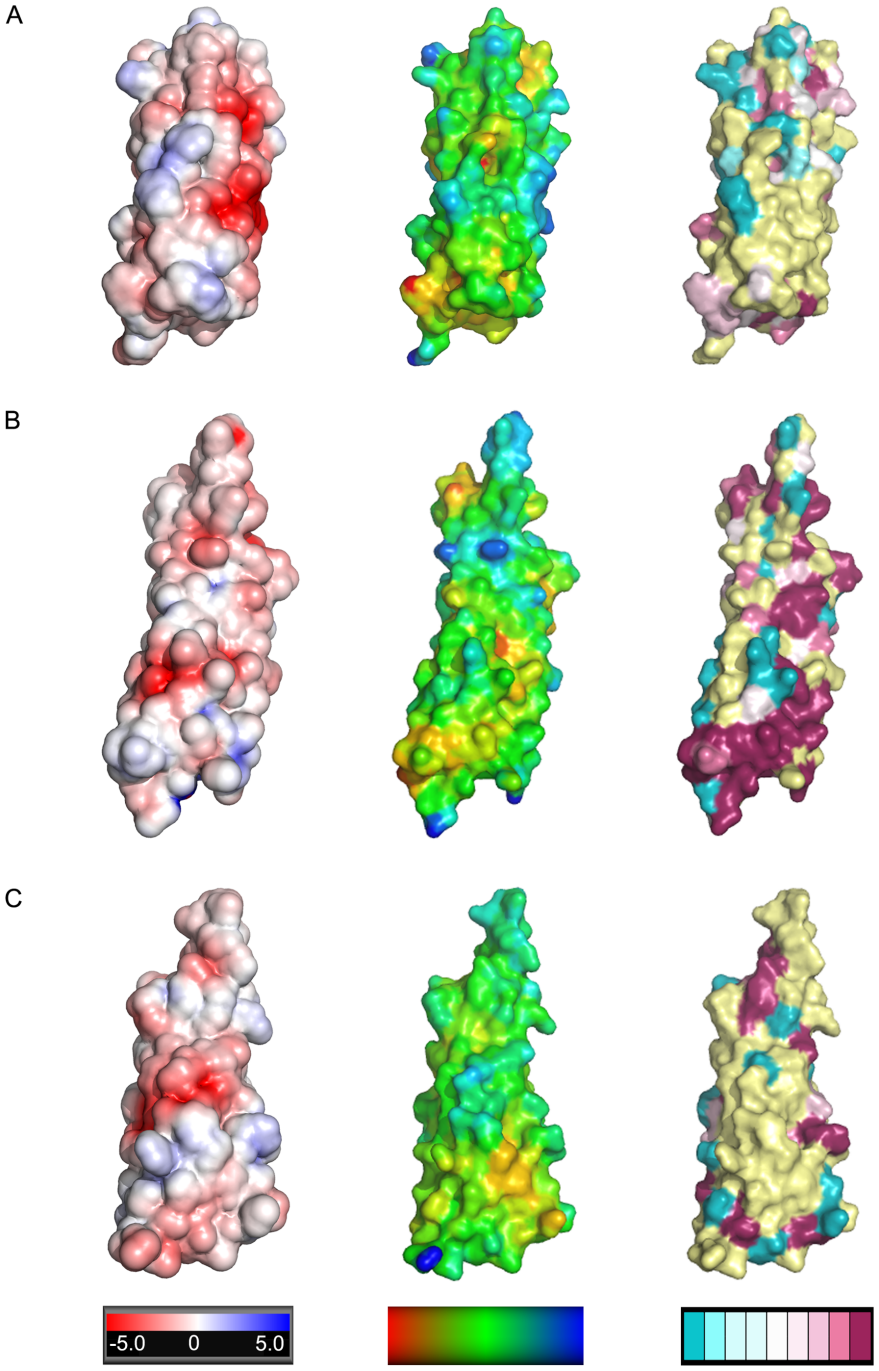
Surface characteristics of Esx complexes (II). View of the Esx complex surfaces with the complexes rotated 180° versus the orientations in Figure 3 to show the CFP-10 homolog side of the complex. With the exception of the rotation the parameters are the same as in Figure 4 with EsxEF_ma_ in (A), EsxGH_ms_ complex in (B), and the EsxOP_mt_ complex in (C).

The contribution of individual Esx complexes to mycobacterial fitness has yet to be fully elucidated, although the EsxAB and EsxGH complexes have been implicated in virulence [58], [63] and metal acquisition [8]–[10], respectively. To elicit clues to the function of the Esx complexes whose structures were determined in this study, we mapped electrostatic charge, hydrophobicity, and sequence conservation on the molecular surfaces of the complexes (Figures 4 and 5). Mapping of charge and hydrophobicity dose not reveal any obvious clues to the function of our Esx complexes as all three complexes show an even distribution of both properties on their surfaces. In contrast, our previous structure of the PE25-PPE41 complex, encoded by *rv2341c*-*rv2340c*, also an ESX secretion substrate, revealed a hydrophobic stripe on the surface of one face of the complex, suggesting a region involved in protein-protein interactions [16]. The mapping of sequence conservation onto the surfaces of the Esx complexes was more informative. The sequence conservation on the surface of the EsxEF_ma_ complex is particularly interesting as the CFP-10 homolog side of the complex shows a high degree of surface variability, while the surface of the ESAT-6 homolog subunit exhibits a diagonal stripe of conserved residues, suggesting a region that may be important for function (Figure 4A). The EsxGH_ms_ complex has a region of significant sequence conservation at the end of the complex that includes the hairpin turn of EsxG_ms_ and the N- and C-termini of EsxH_ms_ (Figure 5B), which correlates with data implicating amino acid residues in this region of the complex in metal binding [9]. At the other end of the EsxGH_ms_ complex there is significant sequence conservation in the N- and C-termini of EsxG_ms_ which both protrude above the core helical bundle structure (Figure 4B). The C-terminus of EsxG_ms_ contains the secretion motif required for complex secretion. Likewise, the C-terminus of EsxP_mt_, which protrudes above the EsxOP_mt_ helical bundle, and which also contains the secretion motif, shows a high degree of sequence conservation (Figure 4C). There are other isolated patches of conserved amino acids on the surfaces of the structures whose functional significance cannot be interpreted in the absence of mechanistic studies exploring the roles of individual amino acid residues in the currently unknown functions of these complexes.

### Comparison with structural homologs

A DALI search of structures deposited in the Protein Data bank was used to find clues to Esx function. However, the lack of unique structural features associated with the helical hairpin of a single CFP-10 or ESAT-6 subunit, or the four-helix bundle of the heterodimeric complex, results in a high degree of structural similarity with many proteins that are unlikely to share any evolutionary or functional overlap with Esx complexes. Nonetheless, the DALI search confirmed that the folds of the EsxEF_ma_, EsxGH_ms_,and EsxOP_mt_ complexes are structurally similar to previously determined Esx complex structures including the homodimeric Esx complexes prevalent in non-mycobacteria (Table 3).

**Table 3:** Structural homologs of the mycobacterial Esx complexes described in this study. Non-redundant targets in the first 200 results of the DALI search results are listed.

Organism (Protein)^a^	PDBid-chain	Z score	RMSD	Number aligned^b^	Sequence identity (%)
**Proteins similar to EsxE_ma_ (PDBid: 4IOX; chain A, MAB_3112)**					
*Bacillus anthracis* str. Sterne (EsxB; homodimer)	4IOE-B	9.8	2.2	75 (82)	21
*M*. *tuberculosis* H37Rv (Rv2430c, PPE41)	2G38-D	8.1	1.9	75 (173)	20
*Staphyloccus aureus* (EsxA; homodimer)	2VS0-B	8.0	2.4	74 (84)	7
*M*. *abscessus* (MAB_3113; ESAT-6 homolog)	4I0X-B	7.7	2.4	74 (83)	22
*Geobacillus thermodenitrificans* (EsxA; homodimer)	3ZBH-D	7.5	2.4	75 (93)	5
*Streptococcus agalactiae* (WXG-100 family protein; homodimer)	3GWK-C	7.4	2.8	73 (98)	14
*M*. *smegmatis* mc^2^155 (MSMEG_0620; CFP-10 homolog)	3Q4H-A	7.4	2.8	73 (90)	14
**Proteins similar to EsxF_ma_ (PDBid: 4IOX; chain B, MAB_3113)**					
*M*. *tuberculosis* H37Rv (Rv3875; ESAT-6)	3FAV-D	9.0	2.7	74 (78)	19
*Helicobacter pylori* (HP0062; homodimer)	3FX7-B	8.8	2.1	77 (87)	16
*M*. *tuberculosis* H37Rv (Rv3874; CFP-10)	3FAV-A	8.7	1.9	72 (74)	19
*M*. *smegmatis* mc^2^155 (MSMEG_0620; CFP-10 homolog)	3Q4H-C	8.3	2.2	75 (87)	13
*B. anthracis* str. Sterne (EsxB; homodimer)	4IOG-C	7.4	3.6	79 (91)	10
*M*. *tuberculosis* H37Rv (Rv2431c, PE25)	2G38-A	7.3	2.4	72 (77)	7
**Proteins similar to EsxG_ms_ (PDBid: 3Q4H; chain A, MSMEG_0620)**					
*B. anthracis* str. Sterne (EsxB; homodimer)	4IOE-B	8.9	2.2	81(82)	16
*M*. *tuberculosis* H37Rv (Rv3875; ESAT-6)	3FAV-D	8.3	3.1	76(78)	14
*M*. *abscessus* (MAB_3113; ESAT-6 homolog)	4I0X-B	8.2	2.3	78(83)	12
*M*. *abscessus* (MAB_3112; CFP-10 homolog)	4I0X-I	8.1	2.2	75(76)	12
*H. pylori* (HP0062; homodimer)	2GTS-A	8.1	2.6	76(77)	11
*S*. *aureus* (EsxA; homodimer)	2VS0-A	8.0	2.9	81(83)	16
*G. thermodenitrificans* (EsxA; homodimer)	3ZBH-B	7.7	3.4	87(92)	9
*M*. *tuberculosis* H37Rv (Rv2430c, PPE41)	2G38-B	6.2	3.6	85 (173)	11
*S. agalactiae* (GBS1074; homodimer)	3O9O-A	6.2	4.1	86 (92)	10
**Proteins similar to EsxH_ms_ (PDBid: 3Q4H; chain B, MSMEG_0621)**					
*M*. *tuberculosis* H37Rv (Rv3875; ESAT-6)	3FAV-D	10.0	1.5	74 (78)	24
*M*. *abscessus* (MAB_3112; CFP-10 homolog)	4I0X-I	8.8	2.8	76 (76)	14
*S*. *aureus* (EsxA; homodimer)	2VS0-A	8.3	2.9	76 (83)	11
*H. pylori* (HP0062; homodimer)	3FX7-B	8.2	2.6	78 (87)	8
*M*. *abscessus* (MAB_3113; ESAT-6 homolog)	4I0X-L	7.9	2.6	77 (82)	8
*B. anthracis* str. Sterne (EsxB; homodimer)	4IOG-D	7.8	2.7	78 (86)	12
*M*. *smegmatis* mc^2^155 (MSMEG_0620; CFP-10 homolog)	3Q4H-C	7.6	3.5	79 (87)	10
*M*. *tuberculosis* H37Rv (Rv2347c; CFP-10 homolog)	3OGI-D	7.5	2.9	70 (74)	11
*M*. *tuberculosis* H37Rv (Rv2430c, PPE41)	2G38-B	7.4	2.8	78 (173)	15
**Proteins similar to EsxO_mt_ (PDBid: 4GZR; chain C, Rv2346c)**					
*M*. *abscessus* (MAB_3113; ESAT-6 homolog)	4I0X-L	5.9	3.3	55 (82)	7
**Proteins similar to EsxP_mt_ (PDBid: 4GZR; chain D, Rv2347c)**					
*S. agalactiae* (GBS1074; homodimer)	3O9O-B	9.3	2.7	78 (96)	13
*G. thermodenitrificans* (EsxA; homodimer)	3ZBH-F	9.1	2.4	78 (93)	17
*S*. *aureus* (EsxA; homodimer)	2VRZ-A	9.0	2.9	78 (98)	10
*M*. *abscessus* (MAB_3112; CFP-10 homolog)	4I0X-I	8.9	1.9	70 (76)	7
*B. anthracis* str. Sterne (EsxB; homodimer)	4IOG-B	8.8	3.0	77 (93)	19
*M*. *smegmatis* mc^2^155 (MSMEG_0621; ESAT-6 homolog)	3Q4H-B	7.5	2.9	74 (80)	11
*M*. *tuberculosis* H37Rv (Rv2431c, PE25)	2G38-A	7.4	2.0	69 (77)	12

a In addition to protein name whether the structurally similar chain is of a CFP-10 or ESAT-6 homolog is noted. Alternatively, if the similar structure is of a homodimeric Esx complex or of a PE protein this information is provided.

b The number in parentheses is the total length of the similar structure.

**Figure 6:**
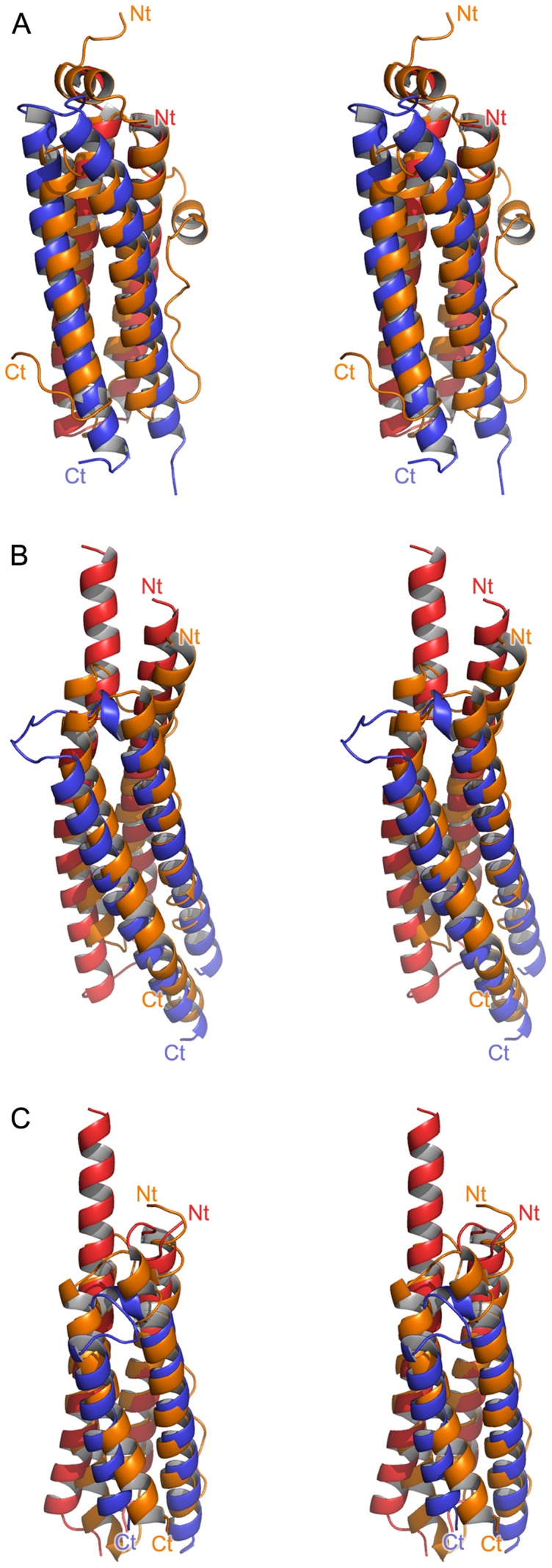
Structural similarity of mycobacterial Esx complexes to ferritin-like proteins. The Esx complexes are oriented and colored as in Figure 3. For clarity only the N-termini of the CFP-10 homologs and C-termini of the ESAT-6 homologs are labeled. Ferritin-like proteins are colored orange and N- and C-termini are labeled. (A) Stereo view of the superposition of EsxEF_ma_ with a single subunit of the *Sulfolobus solfataricus* DPS-like dodecamer assembly (PDBid 2CLB, chain A; Z-score of 10.8 with an RMSD of 2.7 Å for the superposition of 126 amino acids with a sequence identity of 5%). (B) Stereo view of the superposition of EsxGH_ms_ complex with a single subunit of the *E. coli* YciE ferritin-like dimer (PDBid 3OGH, chain A; Z-score of 10.5 with an RMSD of 3.0 Å for the superposition of 134 amino acids with a sequence identity of 6%). (C) Stereo view of the superposition of EsxOP_mt_ complex with a single subunit of the *Bacillus anthracis* BA_0993 hypothetical ferritin-like protein dodecamer (PDBid 2QQY, chain A; Z-score of 9.6 with an RMSD of 3.0 Å for the superposition of 114 amino acids with a sequence identity of 4%).

A DALI search, performed using the four helix bundle of the complex as a single chain search model, reveals that the Esx complexes have a high degree of structural similarity with the four helix bundle proteins of the ferritin superfamily (Figure 6 and Table 4). The data implicating Esx complexes in metal acquisition, specifically EsxGH, and the structural similarity between the Esx bundles and those of ferritin suggest a possible evolutionary relationship. The head-to-tail arrangement of the CFP-10 and ESAT-6 homologs of Esx complexes results in the helices of the Esx complexes having the same topology as those of the ferritin-like proteins. Helices 2 and 3 of ferritin-like protein bundles are connected by a long flexible loop. A similar loop joining the C-terminus of the CFP-10 homolog with the N-terminus of the ESAT-6 homolog would effectively generate a four-helix Esx complex bundle with an architecture similar to the ferritin-like helical bundles. However, a sequence and structural comparison of EsxGH_ms_ with various ferritins (data not shown) failed to identify the canonical ferridoxidase iron binding center and potential metal binding sites on the EsxGH_ms_ surface. Moreover, most ferritins assemble into spherical oligomeric structures of 12 or 24 subunits or head-to-tail dimers [64] and to date the only oligomeric state of an Esx complex greater than a heterodimer has been a domain swapped tetramer [26], which does not resemble ferritin-like dimer molecules. Nonetheless, the overall structural similarity and possibility of a gene fusion or fission event leading to the creation of one protein family from the other is intriguing.

**Table 4:** Top five highest non-redundant results for the DALI search using the two chains of the Esx complexes as a single chain search model.

Rank	Organism; Protein name; PFAM family/superfamily; oligomeric state^a^	PDBid-chain	Z score	RMSD	Number aligned^b^	Sequence identity (%)
**Proteins similar to EsxEF_ma_ (PDBid: 4IOX; chains A and B, MAB_3112-MAB_3113)**						
1	*Sulfolobus solfataricus*; DPS-like protein; ferritin/ferritin-like superfamily; dodecamer	2CLB-A	10.8	2.7	126 (169)	5
2	*Halobacterium salinarum*; DpsA, DPS-like protein; ferritin/ferritin-like superfamily; dodecamer	1TK6-C	10.6	2.8	128 (175)	12
3	*Salmonella enterica*; YciF, bacterial stress protein; domain of unknown function (DUF892)/ ferritin-like superfamily; dimer	4ERU-B	10.6	2.7	129 (158)	9
4	*Treponema pallidum*; antigen TpF1; ferritin/ferritin-like superfamily; dodecamer	2FJC-N	10.6	2.6	121 (150)	11
5	*Xanthomonas campestris*; hypothetical protein XCC3681; domain of unknown function (DUF892)/ferritin-like superfamily; unknown	3HIU-A	10.6	3.0	131 (142)	7
**Proteins similar to EsxGH_ms_ (PDBid: 3Q4H; chains A and B, MSMEG_0620-MSMEG_0621)**						
1	*Escherichia coli*; YciE; domain of unknown function (DUF892)/ferritin-like superfamily; dimer	3OGH-A	10.5	3.0	134 (145)	6
2	*Xanthomonas campestris*; hypothetical protein XCC3681; domain of unknown function (DUF892)/ferritin-like superfamily; unknown	3HIU-B	10.2	3.0	135 (146)	7
3	*Halobacterium salinarum*; DpsA, DPS-like protein; ferritin/ferritin-like superfamily; dodecamer	1TK6-B	9.7	3.4	132 (175)	8
4	*Escherichia coli*; YciF; domain of unknown function (DUF892)/ferritin-like superfamily; dimer	2GS4-A	9.2	3.1	128 (159)	16
5	*Thermosynechococcus elongatus*; DpsA; ferritin/ferritin-like superfamily; dodecamer	2VXX-B	8.9	3.5	131 (173)	8
**Proteins similar to EsxOP_mt_ (PDBid: 4GZR; chains C and D, Rv2346c-Rv2347c)**						
1	*Bacillus anthracis*; hypothetical protein BA_0993; ferritin/ferritin-like superfamily; dodecamer	2QQY-A	9.6	3.0	114 (139)	4
2	*Leptospirillum rubarum*; hypothetical protein LFML04_1845; no PFAM assigned; dimer	3M6J-B	9.1	2.6	96 (126)	3
3	*Deinococcus radiodurans*; hypothetical protein DR_1099; domain of unknown function (DUF305)/ferritin-like superfamily; monomer	3BT5-A	9.0	3.3	115 (151)	10
4	*Xanthomonas campestris*; hypothetical protein XCC3681; domain of unknown function (DUF892)/ferritin-like superfamily; unknown	3HIU-A	9.0	3.1	112 (142)	10
5	Thermosynechococcus elongatus; Dps family DNA-binding stress response protein; ferritin/ferritin-like superfamily; dodecamer	2C41-L	8.9	2.9	112 (155)	9
7	*Mycobacterium tuberculosis*; bacterioferritin (BfrA, Rv1876); ferritin/ferritin-like superfamily; 24 subunits	2WTL-F	8.9	3.0	111 (161)	6

a If known.

b The number in parentheses is the total length of the similar structure.

## Conclusion

Our results demonstrate that MBP fusions are efficient means for the production of mycobacterial Esx protein complexes in *E*. *coli.* The approach is efficient in that expression of the two subunits from a single bicistronic transcript facilitates cloning, and small-scale affinity purification of complexes, coupled with intracellular TEV processing of the fusion protein, provides a rapid assessment of whether the complex is soluble and reconstituted in the absence of MBP. Because obtaining soluble protein is a major bottleneck in structural studies, this approach is useful as either a first approach for expression of protein complexes or, alternatively, as a salvage pathway when complexes fail to express using other methods.

## Supporting Information

Table S1 **Primers used for cloning of Esx complexes.**
(DOCX)

Table S2 **Identifiers of ESAT-6 and CFP-10 protein homologs used to generate sequence alignments for ConSurf calculations (** **Figures 4** **and** **5** **– main text).**
(DOCX)

Table S3 **Length and sequence of intergenic region between CFP-10 and ESAT-6 homolog pairs examined in this study.**
(DOCX)

Table S4 **Summary of small-scale affinity purification of Esx complexes.**
(DOCX)
