# Supplementary material for: Heterologous Expression of Mycobacterial Esx Complexes in Escherichia coli for Structural Studies Is Facilitated by the Use of Maltose Binding Protein Fusions
Source: PLoS One. 2013 Nov 29;8(11):e81753. doi: 10.1371/journal.pone.0081753 (PMC3843698; doi:10.1371/journal.pone.0081753)
Supplement: Table S1 — Primers used for cloning of Esx complexes. (DOCX) [file pone.0081753.s001.docx]

**Table S1.** Primers used for cloning of Esx complexes.

| Primer name | Sequence (5’ - 3’) | Vector |
| --- | --- | --- |
| PIPE.Vec.For. | CGCGACTTAATTAACTCGTTTAAACGGTCTCCAGC | *N/A |
| PIPE.Vec.Rev. | CTGGAAGTACAGGTTTTCGTGATGATGATGATGATG |  |
| Ms0620.For. | AAAACCTGTACTTCCAGGGCATGAGTCTTCTCGACGCTCACATCCC | pMA507/pMA510 |
| Ms0621.Rev. | GAGTTAATTAAGTCGCGTTATCATCCCCACTTGGCGCCTTCG |  |
| Rv2347c.For. | AAAACCTGTACTTCCAGGGCATGGCAACACGTTTTATGACGGATCCGC | pMA507/pMA510 |
| Rv2346c.Rev. | GAGTTAATTAAGTCGCGTTATCAGGCCCAGCTGGAGCCG |  |
| Rv3445c.For. | AAAACCTGTACTTCCAGGGCTTGGTTGAACCAGGAAGGATCGGAG | pMA507/pMA510 |
| Rv3444c.Rev. | GAGTTAATTAAGTCGCGTTACTAACGTGCCCAAGCTCCAGCC |  |
| Rv3905c.For. | AAAACCTGTACTTCCAGGGCATGGGTGCCGACGACACGCTG | pMA507/pMA510 |
| Rv3904c.Rev. | GAGTTAATTAAGTCGCGTTATCACGACCACATACCCAAATTCGTGG |  |
| Ms0620.M3.For. | AACCTGTATTTCCAGAGTATGAGTCTTCTCGACGCTC | pMAPLe3 |
| Ms0621.M3.Rev. | GTGATGGTGATGGTGATGAGTTCCCCACTTGGCGCC |  |
| Rv2347c.M3.For. | AACCTGTATTTCCAGAGTATGGCAACACGTTTTATGAC | pMAPLe3 |
| Rv2346c.M3.Rev. | GTGATGGTGATGGTGATGAGTGGCCCAGCTGGAGC |  |
| Rv3905c.M3.For. | AACCTGTATTTCCAGAGTATGGGTGCCGACGACAC | pMAPLe3 |
| Rv3904c.M3.Rev. | GTGATGGTGATGGTGATGAGTCCACATACCCAAATTCGTG |  |
| Rv3445c.M3.For. | AACCTGTATTTCCAGAGTTTGGTTGAACCGGGAAG | pMAPLe3 |
| Rv3444c.M3.Rev. | GTGATGGTGATGGTGATGAGTGCGTGCCCAAGCTCC |  |
| MAB_0665.For. | AACCTGTATTTCCAGAGTATGACTCCCGCGCGTGACC | pMAPLe4 |
| MAB_0665.Rev. | GTGATGGTGATGGTGATGAGTCCCGGCGATTCCCAGAAAAA |  |
| MAB_3112.For. | AACCTGTATTTCCAGAGTATCGACGAGGTCGGTGC | pMAPLe4 |
| MAB_3113.Rev. | GTGATGGTGATGGTGATGAGTTATCAACCTCGCGCCATTC |  |
| MAB_3754c.For. | AACCTGTATTTCCAGAGTGCTGTTTTTCAGAATGACCTG | pMAPLe4 |
| MAB_3753c.Rev. | GTGATGGTGATGGTGATGAGTTATCAGTGGTGCCAGGCG |  |

*For PCR-amplification of pMA507/pMA510 for SLIC cloning.
