# Supplementary material for: Heterologous Expression of Mycobacterial Esx Complexes in Escherichia coli for Structural Studies Is Facilitated by the Use of Maltose Binding Protein Fusions
Source: PLoS One. 2013 Nov 29;8(11):e81753. doi: 10.1371/journal.pone.0081753 (PMC3843698; doi:10.1371/journal.pone.0081753)
Supplement: Table S2 — Identifiers of ESAT-6 and CFP-10 protein homologs used to generate sequence alignments for ConSurf calculations ( Figures 4 and 5 – main text). (DOCX) [file pone.0081753.s002.docx]

**Table S2.** Identifiers of ESAT-6 and CFP-10 protein homologs used to generate sequence alignments for ConSurf calculations (Figures 4 and 5 – main text).

| Organism | CFP-10 homolog GI (locus tag) | ESAT-6 homolog GI (locus tag) |
| --- | --- | --- |
| **EsxEF_ma_** | | |
| *Mycobacterium abscessus* ATCC 19977 | 169630194 (MAB_3112) | 169630195 (MAB_3113) |
| *Nocardia farcinica* IFM 10152 | 54027559 (nfa55850) | 54027560 (nfa55860) |
| *Mycobacterium xenopi* RIVM700367 | 383824453 (MXEN_06521) | 383824452 (MXEN_06516) |
| *Segniliparus rotundus* DSM 44985 | 296392742 (Srot_0308) | 296392743 (Srot_0309) |
| *Segniliparus rugosus* ATCC BAA-974 | 317505879 (HMPREF9336_00089) | 317505878 (HMPREF9336_00088) |
| *Mycobacterium colombiense* CECT 3035 | 342858988 (MCOL_08928) | 342133230 (MCOL_08933) |
| *Corynebacterium glucuronolyticum* ATCC 51867 | 227487061 (HMPREF0294_0211) | 227487060 (HMPREF0294_0210) |
| *Mycobacterium marinum* M | 183985436 (MMAR_5466) | 183985435 (MMAR_5465) |
| *Mycobacterium tuberculosis* H37Rv | 15611041(Rv3905c) | 15611040 (Rv3904c) |
| *Nocardia cyriacigeorgica* GUH-2 | 379706930 (NOCYR_0677) | 379706929 (NOCYR_0676) |
| *M. abscessus* subsp. *bolletii* BD | 418421218 (MBOL_29400) | 418421219 (MBOL_29410) |
| *Nocardia brasiliensis* ATCC 700358 | 407648271 (O3I_035545) | 407648272 (O3I_035550) |
| *Corynebacterium amycolatum* SK46 | 213965007 (CORAM0001_0239) | 213964911 ( CORAM0001_0240) |
| *Mycobacterium canettii* CIPT 140070010 | 433632992 (BN42_90439) | 433632991 (BN42_90438) |
| *S. rugosus* ATCC BAA-974 | 316255834 (HMPREF9336_00089) | 316255833 (HMPREF9336_00088) |
| **EsxGH_ms_** |  |  |
| *Mycobacterium smegmatis* str. MC^2^155 | 118469490 (MSMEG_0620) | 118470541 (MSMEG_0621) |
| *M. tuberculosis* H37Rv | 183980318 (MMAR_0287) | 183980319 (MMAR_0288) |
| *Mycobacterium bovis* AF2122/97 | 1791466 (Mb0295) | 31791467 (Mb0296) |
| *Mycobacterium gilvum* PYR-GCK | 145213414 (Mflv_0324) | 145213413 (Mflv_0323) |
| *Mycobacterium leprae* Br4923 | 221230841 (MLBr_02532) | 221230840 (MLBr_02531) |
| *M. abscessus* ATCC 19977 | 69629315 (MAB_2229c) | 169629314 (MAB_2228c) |
| *M. marinum* M | 183980574 (MMAR_0546) | 183980575 (MMAR_0547) |
| *Mycobacterium avium* 104 | 118465920 (MAV_4866) | 118465386 (MAV_4865) |
| *M. tuberculosis* H37Rv | 41352776 (Rv3020c) | 2791616 (Rv3019c) |
| *Mycobacterium thermoresistibile* ATCC 19527 | 357018872 (KEK_02671) | 357018871 (KEK_02666) |
| *Mycobacterium ulcerans* Agy99 | 118616923 (MUL_1209) | 118616924 (MUL_1210) |
| *Mycobacterium vanbaalenii* PYR-1 | 120401442 (Mvan_0417) | 120401443 (Mvan_0418) |
| Mycobacterium kansasii ATCC 12478 | 240168397 (MkanA1_03742) | 240168398 (MkanA1_03747) |
| *Mycobacterium sp.* MCS | 108802520 (Mmcs_5560) | 108802521 (Mmcs_5561) |
| *Mycobacterium tusciae* JS617 | 374608005 (MyctuDRAFT_0859) | 374608004 (MyctuDRAFT_0858) |
| *Mycobacterium vaccae* ATCC 25954 | 404446245 (MVAC_23345) | 404446244 (MVAC_23340) |
| *M. fortuitum* subsp. fortuitum DSM 46621 | 404419242 (MFORT_02579) | 404419243 (MFORT_02584) |
| *Mycobacterium massiliense* 2B-0626 | 420951794 (MM2B0626_2024) | 420951793 (MM2B0626_2023) |
| *Mycobacterium rhodesiae* NBB3 | 375138599 (MycrhN_1416) | 375138598 (MycrhN_1415) |
| *Mycobacterium phlei* RIVM601174 | 383819064 (MPHLEI_07147) | 383819065 (MPHLEI_07152) |
| *S. rugosus* ATCC BAA-974 | 317506270 (HMPREF9336_00456) | 317506272 (HMPREF9336_00457) |
| **EsxOP_mt_** |  |  |
| *M. tuberculosis* H37Rv | 1781254 (Rv2347c) | 1781253 (Rv2346c) |
| *M. tuberculosis* H37Rv | 7476904 (Rv1792) | 38684063 (Rv1793) |
| *M. tuberculosis* H37Rv | 1869990 (Rv1038c) | 1869991 (Rv1037c) |
| *M. tuberculosis* H37Rv | 1929071 (Rv1197) | 1929072 (Rv1198) |
| *M. tuberculosis* H37Rv | 2105074 (Rv3620c) | 2105075 (Rv3619c) |
| *M. leprae* Br4923 | 221230075 (MLBr_01181) | 221230074 (MLBr_01180) |
| *M. leprae* Br4923 | 221229989 (MLBr_01055) | 221229990 (MLBr_01056) |
| *M. avium* 104 | 118464008 (MAV_2922) | 118463289 (MAV_2921) |
| *M. avium* 104 | 118467092 (MAV_1178) | 118466478 (MAV_1177) |
| *M. marinum* M | 183982681 (MMAR_2674) | 183982682 (MMAR_2675) |
| *M. marinum* M | 183985088 (MMAR_5120) | 183985087 (MMAR_5119) |
| *M. marinum* M | 183984424 (MMAR_4453) | 183984425 (MMAR_4454) |
| *M. marinum* M | 183983634 (MMAR_3654) | 183983633 (MMAR_3653) |
| *M. marinum* M | 183983640 (MMAR_3660) | 183983639 (MMAR_3659) |
| *M. marinum* M | 183983559 (MMAR_3579) | 183983560 (MMAR_3580) |
| *M. marinum* M | 183983643 (MMAR_3663) | 183983642 (MMAR_3662) |
| *M. ulcerans* Agy99 | 118619704 (MUL_4622) | 118619705 (MUL_4623) |
| *M. ulcerans* Agy99 | 118617121 (MUL_1455) | 118617122 (MUL_1456) |
| *M. ulcerans* Agy99 | 118618466 (MUL_3083) | 118618465 (MUL_3082) |
| *M. bovis* AF2122/97 | 31617818 (Mb1067c) | 31617817 (Mb1066c) |
| *M. bovis* AF2122/97 | 33516878 (Mb1820) | 31618572 (Mb1821) |
| *M. bovis* AF2122/97 | 31617979 (Mb1229) | 31617980 (Mb1230) |
| *Mycobacterium intracellulare* ATCC 13950 | 254822554 (MintA_010100021674) | 254822553 (MintA_010100021669) |
| *Mycobacterium parascrofulaceum* ATCC BAA-614 | 296165332 (HMPREF0591_1315) | 296165331 (HMPREF0591_1314) |
| *M. canettii* CIPT 140070010 | 432164304 (BN42_90119) | 432161733 (BN42_20861) |
| *M. rhodesiae* JS60 | 418052561 (MycrhDRAFT_6163) | 418052560 (MycrhDRAFT_6162) |
| *M. xenopi* RIVM700367 | 383823401 (MXEN_01242) | 383823400 (MXEN_01237) |
