# Supplementary material for: Heterologous Expression of Mycobacterial Esx Complexes in Escherichia coli for Structural Studies Is Facilitated by the Use of Maltose Binding Protein Fusions
Source: PLoS One. 2013 Nov 29;8(11):e81753. doi: 10.1371/journal.pone.0081753 (PMC3843698; doi:10.1371/journal.pone.0081753)
Supplement: Table S3 — Length and sequence of intergenic region between CFP-10 and ESAT-6 homolog pairs examined in this study. (DOCX) [file pone.0081753.s003.docx]

**Table S3.** Length and sequence of intergenic region between CFP-10 and ESAT-6 homolog pairs examined in this study.

| CFP-10/ESAT-6 homologs | IG length (bp) | IG sequence |
| --- | --- | --- |
| MSMEG_0620-MSMEG_0621 | 34 | TCACCCCGTCTTCTTCACCGAACCAGGAGTTCTC |
| Rv2347c-Rv2346c | 50 | CGTCAGCCGCTGCAGCACAATACTTTTACAAGCGAAGGAGAACAGGTTCG |
| Rv3445c-Rv3444c | 20 | ACCGAGGATCAGCCTCGACT |
| Rv3905c-Rv3904c | 10 | GCCGTTTCGG |
| MAB_0665-MAB_0666 | 22 | CCCCAACAGACAGGACAGAGAA |
| MAB_3112-MAB_3113 | 4 | CTGG |
| MAB_3754c-MAB_3753c | 53 | GCGTCTCGTACTAGTCATCTAAGACATCACATAGCAAAGGGGATTGACCAACT |
