# Supplementary material for: Heterologous Expression of Mycobacterial Esx Complexes in Escherichia coli for Structural Studies Is Facilitated by the Use of Maltose Binding Protein Fusions
Source: PLoS One. 2013 Nov 29;8(11):e81753. doi: 10.1371/journal.pone.0081753 (PMC3843698; doi:10.1371/journal.pone.0081753)
Supplement: Table S4 — Summary of small-scale affinity purification of Esx complexes. (DOCX) [file pone.0081753.s004.docx]

| Protein induction temperature 🡪 | | | 37°C | | | | 18°C | | | |
| --- | --- | --- | --- | --- | --- | --- | --- | --- | --- | --- |
| Lysis/purification buffer* 🡪 | | | 1 | 2 | 3 | 4 | 1 | 2 | 3 | 4 |
| Esx complex | Expression vector | *E*. *coli* expression strain |  | | | |  | | | |
| EsxEF_mt_ | pMA507 | BL21 (DE3) | - | - | N/A | N/A | N/A | | | |
|  | pMA510 | BL21 (DE3) | +/- | - | N/A | N/A |  |  |  |  |
|  | pMAPLe3 | BL21 (DE3) | + | N/A | ++ | ++ |  |  |  |  |
|  |  | Rosetta (DE3) | + | N/A | + | + |  |  |  |  |
| EsxGH_ms_ | pMA507 | BL21 (DE3) | - | + | N/A | N/A |  |  |  |  |
|  | pMA510 | BL21 (DE3) | + | + | N/A | N/A |  |  |  |  |
|  | pMAPLe3 | BL21 (DE3) | +++ | N/A | ++ | + |  |  |  |  |
| EsxOP_mt_ | pMA507 | BL21 (DE3) | - | - | N/A | N/A |  |  |  |  |
|  | pMA510 | BL21 (DE3) | + | + | N/A | N/A |  |  |  |  |
|  | pMAPLe3 | BL21 (DE3) | +++ | N/A | ++ | + |  |  |  |  |
| EsxTU_mt_ | pMA507 | BL21 (DE3) | - | - | N/A | N/A |  |  |  |  |
|  | pMA510 | BL21 (DE3) | +/- | + | N/A | N/A |  |  |  |  |
|  | pMAPLe3 | BL21 (DE3) | - | N/A | - | + |  |  |  |  |
|  |  | Rosetta (DE3) | + | N/A | + | + |  |  |  |  |
| EsxEF_ma_ | pMAPLe4 | BL21 (DE3) | ++ | ++ | ++ | + | + | + | + | + |
|  |  | Rosetta (DE3) | + | + | N/A | + | - | - | + | + |
| EsxGH_ma_ | pMAPLe4 | BL21 (DE3) | ++ | ++ | N/A | + | + | + | + | + |
|  |  | Rosetta (DE3) | + | + | N/A | + | - | - | - | + |
| EsxTU_ma_ | pMAPLe4 | BL21 (DE3) | +++ | +++ | +++ | +++ | +++ | +++ | +++ | +++ |
|  |  | Rosetta (DE3) | +++ | +++ | +++ | +++ | ++ | ++ | ++ | ++ |

**Table S4.** Summary of small-scale affinity purification of Esx complexes. The relative amounts of purified protein complex was estimated by SDS-PAGE analysis: -, no complex purified; +/-, negligible amount of purified complex; +, small amount of purified complex (≤ 2.5 mg/L); ++, medium amount of purified complex (2.5-7.5 mg/L); +++, high amount of purified complex (≥7.5 mg/L).

*Buffer composition

1 = 50 mM HEPES, pH 7.5 or pH 7.8, 300 mM NaCl

2 = 50 mM HEPES, pH 7.5, 100 mM NaCl

3 = 50 mM HEPES, pH 7.5, 1 M NaCl

4 = 50 mM HEPES, pH 7.5, 300 mM NaCl, 0.2% LDAO
